# Synergistic effects of *hmp-2*/β-catenin and *sma-1*/β_H_-spectrin on epidermal morphogenesis in *Caenorhabditis elegans*

**DOI:** 10.17912/micropub.biology.000417

**Published:** 2021-07-13

**Authors:** Sydney Wieberg, Harper Euwer, Anna Gerst, Stephanie L. Maiden

**Affiliations:** 1 Department of Biology, Truman State University, Kirksville, MO, 63501

## Abstract

The F-actin, spectrin, and microtubule cytoskeletons are important mediators of embryonic epidermal morphogenesis in *Caenorhabditis elegans*. While SMA-1/β_H_-spectrin is known to organize actin bundles that connect to cadherin-based adhesions, the role of microtubules in the developing epidermis is not well understood. To determine if the spectrin cytoskeleton also plays a role in organizing epidermal microtubules, we conducted feeding RNA interference of four microtubule-associated protein genes in *sma-1/*β_H_-spectrin null animals. Knockdown of *apr-1*,* unc-33*,* unc-44*, or *cls-1 *in *sma-1(ru18)* homozygotes did not reveal a genetic interaction; however, knockdown of *hmp-2/*β-catenin in *sma-1(ru18) *synergistically increased embryonic lethality and epidermal defects.

**Figure 1. Feeding RNAi reveals a genetic interaction between sma-1/βH-spectrin and hmp-2/β-catenin:**
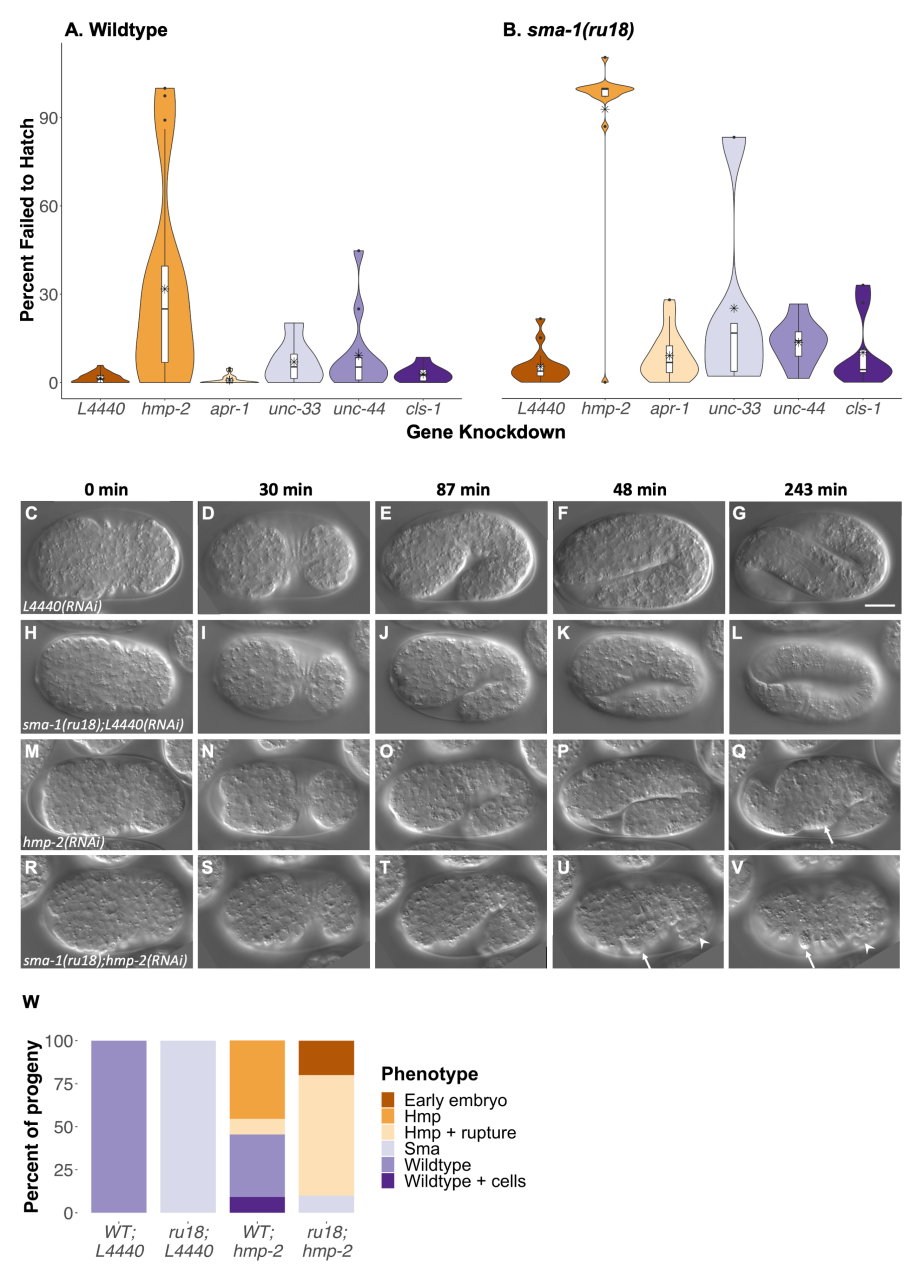
**(A-B)** Percentage of embryos that failed to hatch after feeding RNAi knockdown in wild-type (A) or *sma-1(ru18)* homozygous animals (B). Box limits indicate the 25th and 75th percentiles as determined by R software; whiskers extend 1.5 times the interquartile range from the 25th and 75th percentiles, outliers are represented by dots. Horizontal bars indicate the medians, asterisks indicate the means, and polygons represent density estimates of data and extend to extreme values. From left to right in the plot, n=24, 21, 19, 6, 12, 9, 16, 16, 14, 5, 8, 8 where each *n* is the number of hermaphrodites from which embryos were assessed for failure to hatch. Standard deviations from left to right are 1.66, 33.4, 1.47, 7.59, 13.2, 2.79, 5.76, 25.1, 8.24, 33.4, 7.74, 12.5. **(C-V)** Time-lapse DIC microscopy of wild-type embryos after either empty vector control, *L4440(RNAi)* (C-G), or *hmp-2(RNAi)* (M-Q), and *sma-1(ru18)* homozygous embryos after either *L4440(RNAi)* (H-L) or *hmp-2(RNAi)* (R-V). Arrows = humps, arrowheads = rupture. Embryos are oriented anterior to the left and dorsal up. Scale bar = 10 μm. **(W)** Percent of progeny with specified phenotype observed from DIC data (C-V). From left to right, n=9, 11, 11, 10 where each *n* is a single embryo.

## Description

During embryonic development, changes to cell shape, structure, and location act together to construct an organism’s overall form. Epidermal morphogenesis in *Caenorhabditis elegans* has been a well-studied model for these types of events as this epithelial tissue has substructures like cell-cell and cell-matrix adhesions similar to those in vertebrates (Hsiao and Chisholm 2012). Epidermal cells arise on the dorsal surface of the *C. elegans* embryo with different subsets of cells undergoing various morphogenetic changes to wrap the embryo in an epithelial sheet (Chisholm and Hardin 2005). During dorsal intercalation, two rows of epithelial cells interdigitate with one another to become a single row along the anterior-posterior axis (Priess and Hirsh 1986; Williams-Masson *et al.* 1998). Additional cells from either side of the embryo migrate towards the ventral midline, forming new cell-cell adhesions with their contralateral neighbors (Priess and Hirsh 1986; Williams-Masson *et al.* 1997; Costa *et* *al.* 1998). Contractions along circumferential F-actin bundles in the epidermis then change the shape of the cells to drive elongation of the animal along the anterior-posterior axis (Priess and Hirsh 1986). The spectrin cytoskeleton lies apically between the plasma membrane and circumferential F-actin bundles and is thought to be important in structurally supporting the F-actin network during these contractions (Praitis *et al.* 2005; Lardennois *et al.* 2019). Homozygous mutant animals of *sma-1(ru18)*, a putative null allele of *sma-1*/β_H_-spectrin, are shorter than wildtype with severe disruptions in F-actin bundles normally located at the apical surface (McKeown *et al.* 1998; Praitis *et al*. 2005). Since F-actin bundles are also anchored to cadherin-based adhesions at dorsal-ventral cell boundaries, disruption of HMR-1/cadherin, HMP-2/β-catenin, or HMP-1/α-catenin also results in elongation defects and a loss of F-actin at adherens junctions, as well as embryonic lethality (Costa *et al.* 1998).

Microtubules are organized in a similar pattern to F-actin in the embryonic epidermis and temporally-controlled experiments have shown that they are also required for proper body elongation (Priess and Hirsh 1986; Quintin *et al.* 2016). It is possible that the spectrin cytoskeleton supports the structure and function of the microtubule network as it does for F-actin, but currently no studies have looked at such a connection. Since microtubules are not only essential for epidermal morphogenesis but also early cell divisions, complete disruption would make it difficult to study an interaction with spectrin. Microtubule-associated proteins (MAPs) control many of the important features of microtubules, from dynamic instability to vesicular trafficking (Alfaro-Aco and Petry 2015), and there are at least 60 conserved MAPs in *C. elegans* (Lacroix *et al.* 2014). If there is an interaction between the spectrin cytoskeleton and microtubules that supports proper epidermal morphogenesis, perhaps this can be revealed by weak disruption of specific MAPs in a sensitized, *sma-1*/β_H_-spectrin null background.

RNA interference (RNAi) by feeding is known to cause a weaker knockdown for many genes when compared to RNAi by injection (Timmons and Fire 1998). To take advantage of this, we conducted feeding RNAi of four MAP genes (*apr-1*, *unc-33*, *unc-44*, *cls-1)* in wild-type and *sma-1(ru18)* homozygous animals and determined the number of progeny that failed to hatch (Fig. 1A-B). In wildtype (Fig. 1A), two-way ANOVA with Tukey comparison found no significant differences in embryonic lethality between the L4440 empty vector RNAi control and any of the four MAP genes tested(*apr-1,* p=1.00; *unc-33*,p=1.00; *unc-44*, p=0.98; *cls-1*, p=1.00). Consistent with previous findings, *sma-1(ru18)* animals fed the L4440 control showed very low levels of embryonic lethality (Fig. 1B) similar to those found in the wild-type L4440 control (Fig. 1A; p=1.00). RNAi of the four MAP genes in *sma-1(ru18)* homozygous animals also showed no significant changes in embryonic lethality when compared to either the *sma-1(ru18)* L4440 control (*apr-1,* p=1.00; *unc-33,* p=0.48; *unc-44,* p=0.99; *cls-1*, p=1.00) or the same treatment in wildtype (*apr-1,* p=0.97; *unc-33,* p=0.83; *unc-44,* p=1.00; *cls-1*, p=1.00). It is important to note that knockdown by feeding RNAi may have been ineffective for these four MAP genes. For example, since roughly 65% of *apr-1*/APC homozygous null animals are embryonic lethal (Hoier *et al.* 2000), one would expect even weak *apr-1* knockdown in wildtype to exhibit some significant lethality. At the very least the results indicate that if these MAPs play a role during epidermal morphogenesis, the *sma-1(ru18)* sensitized background is insufficient to reveal that role.

In contrast, RNAi of *hmp-2*/β-catenin in the *sma-1(ru18)* background did reveal an important genetic interaction during embryogenesis. As a positive control in wild-type animals, *hmp-2(RNAi)* resulted in an average of 31.8% embryos that failed to hatch, which was significantly different than the average 1.5% of embryos that failed to hatch in the RNAi vector control, L4440 (Fig. 1A; p<0.01). In *sma-1(ru18)* homozygous animals, *hmp-2* knockdown resulted in even greater embryonic lethality, with 92.8% of embryos that failed to hatch compared to 5.3% in L4440 (Fig. 1B), a statistically significant increase of 61.0% over the *hmp-2* knockdown in wildtype (p<0.01).

To determine the cause of the increased embryonic lethality in *sma-1(ru18); hmp-2(RNAi)* animals, we used time-lapse differential interference contrast (DIC) microscopy (Fig. 1C-V) and categorized the resulting phenotypes (Fig. 1W). In wild-type control embryos (Fig. 1C-G), epidermal cells properly migrated from the dorsal surface to the ventral midline (Fig. 1C-D) before animals elongated along the body axis (Fig. 1E-G). Consistent with the literature, homozygous *sma-1(ru18)* embryos (Fig. 1H-L) completed ventral enclosure successfully (Fig. 1H-I) but body elongation was stunted (Fig. 1J-L), with 100.0% of embryos reaching only two-fold their original length (Fig. 1L) compared to the four-fold increase seen in wild-type animals (Fig. 1G). In wild-type embryos with *hmp-2* knockdown (Fig. 1M-Q), 45.5% of animals showed elongation defects (Fig. 1O-Q) similar to those of *sma-1(ru18)* homozygotes (Fig. 1J-L) but with additional body morphology defects (i.e., humps) (Fig. 1Q; arrow). In 9.0% of wild-type *hmp-2(RNAi)* embryos, animals appeared to elongate four-fold but extraneous material, possibly cells, was observed in the egg shell (Fig. 1W). In *sma-1(ru18); hmp-2(RNAi)* embryos, 70.0% of embryos elongated to only 1.5-fold their original body length (Fig. 1T-U) before forming humps (Fig. 1U-V; arrow), with each of these embryos exhibiting cell ruptures from the posterior after the onset of humps (Fig. 1U-V; arrowhead). In 20.0% of *sma-1(ru18); hmp-2(RNAi)* embryos, the landmarks of dorsal intercalation or ventral enclosure were not apparent (Fig. 1W; Extended Data), indicative of an earlier defect in embryogenesis.

In hindsight, a genetic interaction between *sma-1/*β_H_-spectrin and *hmp-2/*β-catenin could have been predicted based on the roles of these two genes in epidermal morphogenesis. At sites of cell-cell adhesion in the embryonic epidermis, HMP-2/β-catenin acts as a bridge between HMR-1/cadherin and the F-actin binding protein HMP-1/α-catenin (Costa *et al*. 1998; Maiden *et al*. 2013). Loss of HMP-2 therefore results in a complete loss of F-actin at the adherens junction (Costa *et al*. 1998), whereas *sma-1(ru18)* homozygous mutants maintain F-actin at adherens junctions but the circumferential actin bundles are disorganized (Praitis *et al.* 2005). Even though *hmp-2* and *sma-1* perturbation both disrupt epidermal F-actin, our expectation was that the embryonic lethality observed in *hmp-2(RNAi)* embryos would simply supersede the mild elongation defects in *sma-1(ru18)* homozygous mutants and that the percent of embryos that failed to hatch in *sma-1(ru18)*; *hmp-2(RNAi)* would be similar to *hmp-2(RNAi)* in wild-type animals. The earlier formation of humps and the cells rupturing from the posterior in *sma-1(ru18); hmp-2(RNAi)* embryos, however, would indicate that the genetic interaction between *sma-1* and *hmp-2* is important in maintaining stable cell-cell adhesions due to the roles of each in proper F-actin attachment and organization. However, the 20.0% of embryos that appeared to have defects earlier than dorsal intercalation or ventral enclosure may be evidence of a role for these two genes in the cell shape changes occurring during gastrulation. As observed in the embryonic epidermis, SMA-1 may be required to properly organize and maintain actin bundles along the apical membrane surface in cells undergoing apical constriction, like the endoderm precursor cells (Goldstein and Nance 2020). HMP-2/β-catenin and HMP-1/α-catenin have already been shown to be required for HMR-1/cadherin to become more apically enriched at lateral contacts between these endoderm precursor cells during gastrulation (Marston *et al.* 2016), so disruption of both *sma-1* and *hmp-2* could more severely perturb the organization of actin and cadherin-based adhesions in these cells. A role for β_H_-spectrin in gastrulation has already been found in *Drosophila* ventral furrow invagination, where a loss of β_H_-spectrin prevents stabilization of the cell surface during the pulsatile events of apical constriction (Krueger *et al*. 2020). A similar but mechanically distinct ratcheting mechanism has been proposed for *C. elegans* SPC-1*/*α-spectrin in the muscle-dependent stage of embryonic elongation (i.e., past two-fold) (Lardennois *et al*. 2019). Further mechanistic studies will be needed to determine the potential role of SMA-1/β_H_-spectrin and HMP-2/β-catenin during *C. elegans* gastrulation.

## Methods

*C. elegans* strains were cultured using standard protocols (Brenner 1974). Strains used include N2 [wildtype] and AZ30 [*sma-1(ru18)*].

Bacterial clones for feeding RNAi were obtained from the Ahringer library (Kamath and Ahringer 2003). The *hmp-2* gene insert was verified by Sanger sequencing through Eurofins Genomics using the following vector primers: GTCAGTGAGCGAGGAAGCAAC and CTCTTCGCTATTACGCCAGCTG. For knockdown by feeding RNAi, bacterial cultures were plated on NGM plates supplemented with 25 μg/mL carbenicillin, 10 μg/mL tetracycline, and 1 mM IPTG, and incubated for 3 days at room temperature to induce double-stranded RNA. L4 worms at 20 °C were fed bacteria for 48 hours and then singled to individual plates. After laying eggs for approximately 18 hours, the adults were removed and all progeny were counted via a dissecting microscope. After an additional 24 hours, the remaining number of eggs were counted. The violin plot, basic summary statistics, two-way ANOVA Type III, and the post-hoc Tukey analysis were all completed using R.

For time-lapse DIC microscopy, eggs were collected from gravid hermaphrodites after L4 worms were fed RNAi bacteria for 48 hours. Eggs were mounted on a 5% agarose slide and imaged using 1-μm slice spacing throughout the embryo at 3-minute intervals over 9 hours using a Leica DIC microscope with a 63x/1.25 NA oil HCX PL FLUOTAR objective at 20 °C with a Windows PC computer running IPLab 4.0 software and a Qimaging QICAM Fast 1394 camera. ImageJ was then used to analyze the 4D dataset.
